# p47^phox^-Dependent Oxidant Signalling through ASK1, MKK3/6 and MAPKs in Angiotensin II-Induced Cardiac Hypertrophy and Apoptosis

**DOI:** 10.3390/antiox10091363

**Published:** 2021-08-26

**Authors:** Fangfei Liu, Lampson M. Fan, Li Geng, Jian-Mei Li

**Affiliations:** 1 School of Biological Sciences, University of Reading, Reading RG6 6AS, UK; liufangfei97@gmail.com (F.L.); gengli@jiangnan.edu.cn (L.G.); 2 The Royal Wolverhampton NHS Trust, Wolverhampton WV10 0QP, UK; lampson.fan1@nhs.net; 3 Faculty of Health and Medical Sciences, University of Surrey, Guildford GU2 7XH, UK

**Keywords:** redox-signalling, p47^phox^, knockout mice, Angiotensin II, cardiac hypertrophy, apoptosis

## Abstract

The p47^phox^ is a key regulatory subunit of Nox2-containing NADPH oxidase (Nox2) that by generating reactive oxygen species (ROS) plays an important role in Angiotensin II (AngII)-induced cardiac hypertrophy and heart failure. However, the signalling pathways of p47^phox^ in the heart remains unclear. In this study, we used wild-type (WT) and p47^phox^ knockout (KO) mice (C57BL/6, male, 7-month-old, *n* = 9) to investigate p47^phox^-dependent oxidant-signalling in AngII infusion (0.8 mg/kg/day, 14 days)-induced cardiac hypertrophy and cardiomyocyte apoptosis. AngII infusion resulted in remarkable high blood pressure and cardiac hypertrophy in WT mice. However, these AngII-induced pathological changes were significantly reduced in p47^phox^ KO mice. In WT hearts, AngII infusion increased significantly the levels of superoxide production, the expressions of Nox subunits, the expression of PKCα and C-Src and the activation of ASK1 (apoptosis signal-regulating kinase 1), MKK3/6, ERK1/2, p38 MAPK and JNK signalling pathways together with an elevated expression of apoptotic markers, i.e., γH2AX and p53 in the cardiomyocytes. However, in the absence of p47^phox^, although PKCα expression was increased in the hearts after AngII infusion, there was no significant activation of ASK1, MKK3/6 and MAPKs signalling pathways and no increase in apoptosis biomarker expression in cardiomyocytes. In conclusion, p47^phox^-dependent redox-signalling through ASK1, MKK3/6 and MAPKs plays a crucial role in AngII-induced cardiac hypertrophy and cardiomyocyte apoptosis.

## 1. Introduction

Nicotinamide adenine dinucleotide phosphate oxidase (NADPH oxidase, or Nox) is a membrane-bound enzyme that by generating reactive oxygen species (ROS) plays important role in the regulation of cellular function. So far, seven isoforms of the catalytic component of Nox have been identified namely Nox1–5, and durox 1–2 [1]. Angiotensin II is a vasoconstricting peptide (Asp-Arg-Val-Tyr-Ile-His-Pro-Phe) of the renin-angiotensin-aldosterone system involved in the regulation of blood pressure and other aspects of organ functions [2]. Oxidative stress and inflammation due to the activation of a Nox2-contaninig NADPH oxidase (Nox2) has been found to play an essential role in mediating AngII-induced cardiac hypertrophy and failure [1,2,3,4,5]. Nox2 is a multi-subunit enzyme consisting of two membrane-bound subunits, p22^phox^ and Nox2 (also named gp91^phox^), and four cytosolic regulatory subunits, i.e., p40^phox^, p47^phox^, p67^phox^ and rac1. The p47^phox^ is a key regulatory subunit of Nox2 enzyme [2,6,7]. The phosphorylation of p47^phox^ initiates the process of coordination and association of regulatory subunits with membrane-bound p22^phox^/Nox2 complex, and the subsequent O_2_^●−^ production [2,6].

In the mammalian heart, the p47^phox^ is expressed in the myocardium, epicardium and coronary vessels [1]. In cardiomyocytes p47^phox^ had been reported to co-localise with F-actin and cortactin in order to facilitate the translocation of the cytosolic regulatory subunits to the p22^phox^/Nox2 complex [8,9]. Under pathological conditions, p47^phox^ was suggested to link oxidative stress with the hypertrophic growth of cardiomyocytes through the activation of mitogen-activated protein kinases (MAPKs), i.e., extracellular signal-regulated kinase (ERK), c-Jun N-terminal kinase (JNK) and p38 mitogen-activated protein kinase (p38MAPK) [10,11]. In response to oxidative stress, the redox-sensitive MAPK kinase (MKK) to MAPKs signalling pathways are activated, which in turn promote the activities of pro-apoptotic signalling molecules such as p53, γH2AX and apoptosis signal-regulating kinase 1 (ASK1) leading to cardiac damage [12,13,14]. Genetic ablation of p47^phox^ attenuated AngII-induced abdominal aortic aneurysm formation in apolipoprotein E-deficient mice [15], and reduced the level of cardiac hypertrophy after experimental myocardial infarction [16].

Despite the importance of p47^phox^ as a key regulator of Nox2-derived ROS production in the heart, its signalling pathways and functional complexity in AngII-induced cardiac hypertrophy and cardiomyocyte damage remained unclear. There was insufficient information about the upstream and downstream signalling pathways of p47^phox^ in the hearts. In the current study, we investigated the role of p47^phox^ and its oxidant-signalling pathways in the hearts using a murine model of AngII-infusion-induced cardiac hypertrophy and cardiomyocyte apoptosis in WT and p47^phox^ KO mice. The complex role of p47^phox^ in the myocardium was investigated by examining AngII-induced cardiac oxidative stress, the expressions of Nox subunits, the expression of PKCα and C-Src (both were involved in p47^phox^ phosphorylation). We also examined the levels of AngII-induced p47^phox^ phosphorylation, the activation of redox-sensitive ASK1, MKK3/6 and MAPKs and the expression of pro-apoptotic markers, i.e., γH2AX and p53 in the cardiomyocytes. Our results suggested that p47^phox^ oxidant-signalling through ASK1, MKK3/6 and MAPKs played a vital role in mediating cardiac hypertrophic response and the expression of apoptotic markers in cardiomyocytes in response to AngII challenge. Knockout of p47^phox^ inhibited the activation of these stress signalling pathways and protected hearts from AngII-induced oxidative damages.

## 2. Materials and Methods

### 2.1. Chemicals and Reagents

AngII was purchased from Sigma-Aldrich (Amersham, UK); NADPH was purchased from Fisher Scientific (Loughborough, UK); dihydroethidium (DHE) was purchased from Invitrogen (Loughborough, UK); FITC-labelled wheat germ agglutinin (WGA, Catalogue No. L-4895) was from Sigma-Aldrich. Primary antibodies to p47^phox^, p22^phox^, Nox1, Nox2, Nox4, p38-MAPK, ERK1/2, phos-JNK (Thr183/Tyr185) and total JNK, phos-Akt (Ser473) and total Akt were purchased from Santa Cruz Biotechnology (Dallas, TX, USA); antibodies to β-actin, phos-MKK3(Ser189)/6(Ser207) and phos-ASK1 (Thr845), total MKK3/6, γH2AX (Ser139/Tyr142) were purchased from Cell Signalling Technology (London, UK); Antibodies to phos-p47^phox^ (Ser359), phos-p38-MAPK (Thr180/Tyr182) and phos-ERK1/2 (Thr202/Tyr204) were purchased from Sigma-Aldrich. Nox2-ds-tat (Nox2tat, [H]-RKKRRQRRRCSTRVRRQL-[NH2]) were provided by PeptideSynthetics (PPR Ltd., Fareham, UK). Other reagents, chemicals and antibodies, unless specified, were purchased from Sigma-Aldrich.

### 2.2. Animals

All studies were performed following protocols approved by the Ethics Committees of the Surrey and Reading Universities and the Home Office under the Animals (Scientific Procedures) Act 1986 UK. The p47^phox^ KO mice on a 129sv background were initially obtained from the European mouse mutant archive, and backcrossed to C57BL/6 for ten generations at the animal units in the University of Surrey [17]. Littermates of wild-type and p47^phox^ KO mice at the age of 7-months were randomly grouped into control and AngII groups (*n* = 9 per group). The dose of AngII (0.8 mg/kg/day) was chosen based on the literature and our own pilot experiments to produce significant cardiac hypertrophy effectively. AngII was delivered to mice through osmotic minipumps (ALZET osmotic pumps, DURECT Corporation, Cupertino, CA, USA) for 14 days. The control group was infused with saline. Systolic and diastolic blood pressure (BP) were measured using a computerised tail-cuff system (CODA, Kent Scientific, Torrington, CT, USA) on conscious mice following one week of training with the instrument [18]. Mice were used at the end of two weeks of AngII infusion. Bodyweight and heart weight were measured, and the tissues were harvested and stored in −80 °C freezer for experimental use.

### 2.3. Measurement of Cross-Sectional Cardiomyocyte Sizes 

Left ventricular cryosections (8 µm) were prepared and fixed in freshly prepared 1% formaldehyde phosphate-buffered solution. Cardiomyocytes in the cardiac sections were outlined by FITC-conjugated wheat germ agglutinin (WGA) that binds to glycoproteins of the cell membrane, and is routinely used for the staining of cardiac sarcolemma to determine cross sectional area or myocyte density [19]. Staining was visualised under the A1R confocal microscope (Nikon, Chiyoda, Japan) (20–40× magnification, 1024 × 1024 pixels)). Cross-sectional cardiomyocyte sizes were measured according to the method published previously [19,20] using software of ImageJ 1.50i (NIH, Bethesda, MD, USA). For statistical analyses, cardiomyocyte sizes were obtained from at least three microscopic areas per section, three cross sections per heart and nine mice per group. 

### 2.4. Measurement of ROS Production

ROS production was measured using the homogenates of left ventricular tissues. The homogenates were used immediately for the ROS measurement as described previously using three independent methods [4]. Lucigenin (5 µM)-chemiluminescence was used for measuring real-time NADPH-dependent O_2_^●−^ production in heart homogenates detected using a 96-well microplate luminometer (Molecular Devices, Wokingham, UK). Catalase (300 U/mL)-inhibitable amplex red (6.25 µM) assay was used for measuring the H_2_O_2_ production in heart homogenates detected using FluoStar OPTIMA (BMG LabTech, Aylesbury, UK). DHE (2 µM)-fluorescence was used to measure in situ ROS production by cardiac sections, and images were captured using Nikon Eclipse Ti2-E inverted microscope and the DHE fluorescence intensities were quantified. The specificity of the lucigenin and DHE assays for the detection of O_2_^●−^ was confirmed by using tiron (10 mM), a non-enzymatic O_2_^●−^ scavenger, and superoxide dismutase (SOD) (200 U/mL). The enzymatic sources of O_2_^●−^ production were investigated using different enzyme inhibitors, i.e., L-NAME (N-nitroarginine methyl ester, 100 μM, nitric oxide synthase inhibitor), rotenone (100 μM, mitochondrial complex-1 enzyme inhibitor), diphenyleneiodonium (DPI) (20 μM, flavoprotein inhibitor), oxypurinol (100 μM, xanthine oxidase inhibitor) and Nox2tat (a specific peptide Nox2 inhibitor, 10 µM) [21]. Individual inhibitor was added into the wells loaded with homogenates and incubated for 10 min at room temperature before the measurement of ROS production.

### 2.5. Immunoblotting

Immunoblotting was performed exactly as described previously [4,18] using the left ventricular tissue homogenates. β-actin detected in the same sample was used as a loading control. For the quantification of phosphorylation of MAPKs, the total levels of the same protein in the same sample were pre-tested and justified for equal loading and used as loading controls for the quantification of phosphorylated proteins. The results were captured by BioSpectrum AC imaging system (UVP, Upland, CA, USA). The optical density of the bands was quantified and normalised to the relevant loading controls. 

### 2.6. Immunofluorescence Microscopy 

These experiments were performed as described previously [18]. The left ventricular tissue cryosections (8 µm) were fixed with 1:1 methanol: acetone solution for 10 min at −20 degree. All buffers and reagents were freshly prepared and kept on ice before use. Sections were then blocked using 2% bovine albumin serum (BSA) in PBS with 0.1% Triton X-100. BSA (2%) was used in the place of primary antibodies as a negative control. Primary antibodies were used at 1:100 dilutions in 0.2% BSA/PBS. Biotin-conjugated secondary antibodies were used at 1:1000 dilution in 0.2% BSA/PBS and detected using streptavidin-FITC or streptavidin-Cy3. Images were captured by Nikon A1R confocal microscope, and the fluorescent intensities (Fluo-intensity) were quantified. For statistical analysis, at least five random fields per section with three sections per heart were used per animal and nine animals were used per group. The control background fluorescence captured from sections without primary antibody was deducted and the results were expressed as Fluo-intensity. 

### 2.7. Statistics

The Statistical analysis was performed using GraphPad Prism 7.0. Two-way ANOVA plus Tukey’s multiple comparison test were used for multiple-group significance testing and for testing repeated measures of blood pressure. One-way ANOVA followed by a Bonferroni post-hoc test was employed for other data analyses where it was appropriate. *p* ≤ 0.05 was denoted as statistically significant. Nine mice per group were used for statistical analysis. Results were presented as mean ± SD unless specified in the figure legends.

## 3. Results

### 3.1. Knockout p47^phox^ Attenuated AngII Infusion-Induced High Blood Pressure and Cardiac Hypertrophy

**Figure 1:**
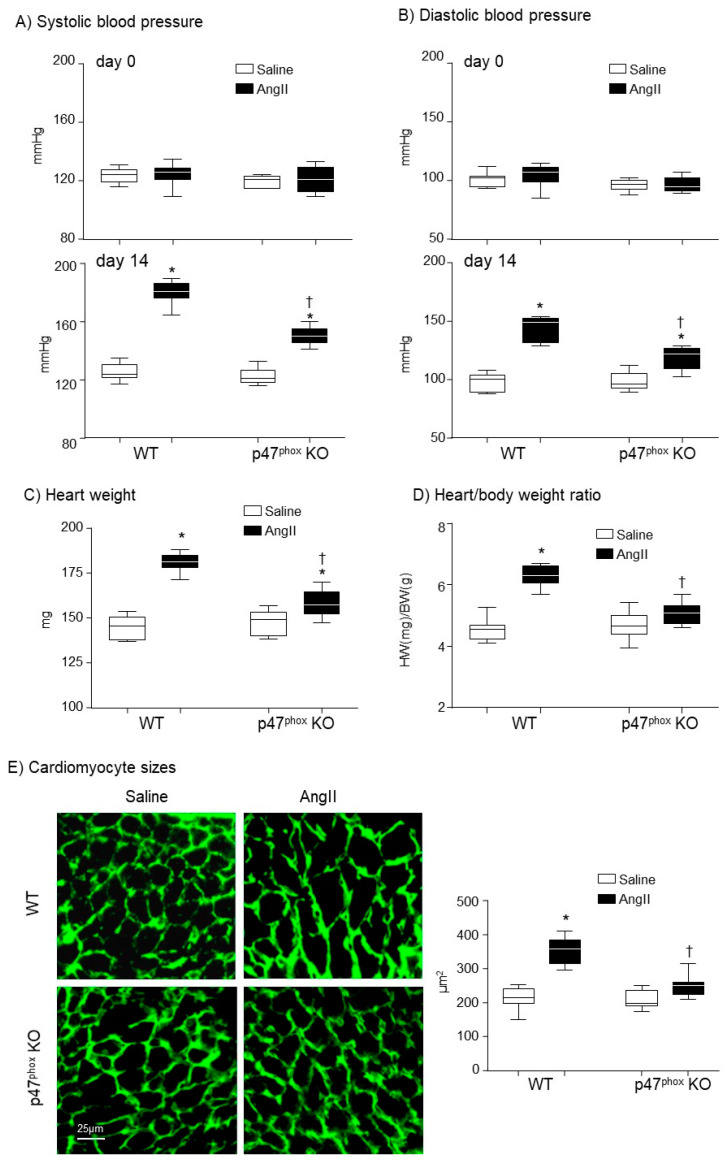
Development of hypertension and cardiac hypertrophy in AngII-infused WT and p47^phox^ KO mice. (**A**) Systolic blood pressure. (**B**) Diastolic blood pressure. Day 0: day of minipump implantation. Day 14: day of minipump removal. (**C**) Heart weights. (**D**) Heart weight (HW, mg)/body weight (BW, g) ratio. (**E**) Left panels: Representative images of cardiomyocyte sizes on the cross-sections of left ventricular tissues. The cardiomyocytes membranes were labelled with WGA-FITC (green). Right panel: Statistical analysis of cardiomyocyte cross-sectional areas (µm^2^). *n* = 9 mice per group. Statistical analyses were performed using two-way ANOVA. * *p* < 0.05 for AngII values versus saline values in the same genetic group; ^†^ *p* < 0.05, for p47^phox^ KO AngII values versus WT AngII values.

The mice used in this study were middle-aged (7-months) which were more susceptible to AngII-induced cardiovascular damages than mice at younger ages. At day 0 (before AngII challenge), there was no significant difference in BP between WT and p47^phox^ KO mice. AngII infusion (14 days) of WT mice markedly increased the systolic BP to an average of 180.3 ± 7.5 mmHg and the diastolic BP to an average of 142.6 ± 10.8 mmHg as compared to saline-infused controls (Figure 1A,B). However, in the absence of p47^phox^, AngII infusion only caused mild but significant increases in the systolic BP to an average of 150 ± 6 mmHg and the diastolic blood pressure to an average of 118.6 ± 9.7 mmHg (Figure 1A,B). The levels of AngII-induced cardiac hypertrophy were expressed as the increases in heart weight (HW) and the HW/body weight (BW) ratios. In WT mice, AngII infusion significantly increased the heart weights (Figure 1C) and the HW/BW ratios (Figure 1D). However, in the p47^phox^ KO mice, AngII induced cardiac hypertrophy was significantly reduced in comparison to WT mice. Although there were increases in HW/BW ratio in AngII-infused p47^phox^ KO mice, these were not statistically significant (Figure 1D). AngII-induced cardiac hypertrophy was further examined by measuring cardiomyocyte cross sectional area in the left ventricular tissue sections. The cardiomyocytes were labelled with FITC-WGA, which binds to glycoproteins of the cardiomyocyte membrane and outlines the cardiomyocytes on cross sections [19]. In comparison to saline-infused controls, there were significant increases in the cross-sectional areas of cardiomyocytes in AngII-infused WT hearts, which were significantly reduced in p47^phox^ KO hearts (Figure 1E).

### 3.2. Knockout p47^phox^ Inhibited AngII-Induced Cardiac Oxidative Stress

**Figure 2:**
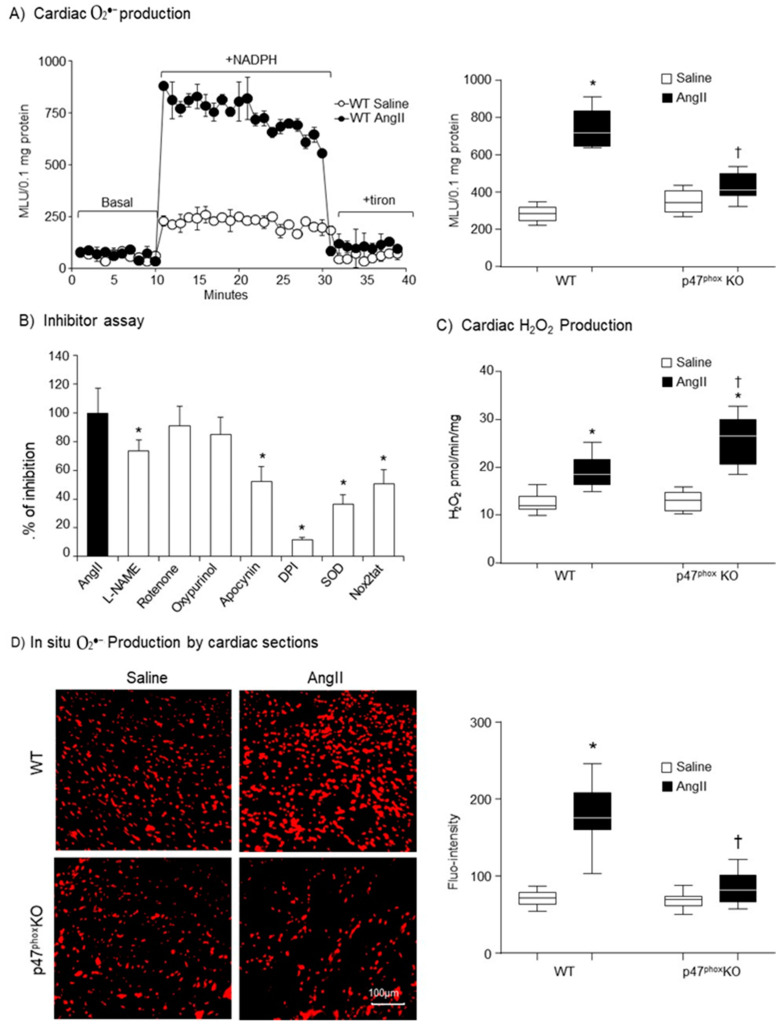
Cardiac ROS production. (**A**) Levels of O_2_^●−^ production measured by lucigenin-chemiluminescence. Left panel: Representative examples of kinetic measurements of O_2_^●−^ production by WT heart homogenates. NADPH (0.1 mM) was added at 10 min. Tiron (10 mM) was added at 30 min to scavenge O_2_^●−^. Right panel: Differences in NADPH-dependent O_2_^●−^ production measured between 10–30 min shown in the left panel. (**B**) The effects of different enzyme inhibitors on the levels of O_2_^●−^ by AngII-infused WT heart homogenates. L-NAME: (NG-nitroarginine methyl ester), NOS inhibitor; Rotenone: mitochondrial respiratory chain inhibitor; Oxypurinol, xanthine oxidase inhibitor; DPI: (diphenyleneiodonium), flavoprotein inhibitor; SOD: (superoxide dismutase). (**C**) Cardiac H_2_O_2_ production detected by amplex red assay. (**D**) In situ detection of reactive oxygen species production by DHE fluorescence. Left panel: Representative images of DHE staining on cardiac sections; right panel: Quantification of DHE fluorescence intensity. *n* = 9 per group. Statistical comparisons were done using one-way ANOVA for inhibitor assay, and two-way ANOVA for the rests (panels A, C, D). * *p* < 0.05 for indicated AngII values versus saline values in the same genetic group; ^†^ *p* < 0.05 for p47^phox^ KO AngII values versus WT AngII values.

The effect of genetic ablation of p47^phox^ on AngII-induced cardiac oxidative stress were first examined by measuring NADPH-dependent O_2_^●−^ production in heart homogenates using lucigenin chemiluminescence. A representative example of real-time measurements of O_2_^●−^ production by heart homogenates is shown in the left panel of Figure 2A. Tiron (an O_2_^●−^ scavenger) was used to confirm the assay specificity. The statistical analyses were shown in the right panel of Figure 2A. Compared to saline-infused WT controls, AngII infusion resulted in 2.6-folds increases in the levels of O_2_^●−^ production in the WT hearts. However, this was significantly inhibited by knockout of p47^phox^. Although there were some increases in the levels of O_2_^●−^ production in AngII infused p47^phox^ KO hearts, they were not statistically significant. The enzymatic sources of AngII-induced O_2_^●−^ production found in WT hearts were examined using different enzyme inhibitors including L-NAME (nitric oxide synthase inhibitor), rotenone (mitochondrial respiratory chain inhibitor), oxypurinol (xanthine oxidase inhibitor), apocynin (NADPH oxidase inhibitor), DPI (flavoprotein inhibitor) and Nox2tat (a specific peptide inhibitor of Nox2) (Figure 2B). The O_2_^●−^ production detected in AngII-infused WT hearts was not affected by rotenone and oxypuronol, but was significantly inhibited by apocynin, Nox2tat or DPI suggesting Nox2 as a major enzymatic source of AngII-induced O_2_^●−^ production. There was some inhibition of AngII-induced O_2_^●−^ production by L-NAME, indicating nitric oxide synthase disfunction. SOD (superoxide dismutase) was used to double confirm the detection of O_2_^●−^. 

O_2_^●−^ is not stable and can be quickly converted to H_2_O_2_ in cells. Therefore, we examined cardiac H_2_O_2_ production using catalase-inhibitable amplex red assay (Figure 2C). There was no significant difference in the basal (without AngII) levels of H_2_O_2_ production between WT and p47^phox^ KO hearts. Compared to saline infused controls, the level of H_2_O_2_ production was significantly increased in AngII-infused WT hearts, which might link to the high level of O_2_^●−^ production. However, in the p47^phox^ KO hearts, although the level of O_2_^●−^ production showed no change in response to AngII challenge, there was a significant increase in the levels of H_2_O_2_ production in AngII-infused p47^phox^ KO hearts as compared to saline controls.

The levels of AngII-induced O_2_^●−^ production in the hearts were further examined by in situ DHE fluorescence on cardiac cryosections (Figure 2D). There were significant high levels of O_2_^●−^ production in AngII-infused WT hearts in comparison to saline-infused WT controls. However, there was no significant difference in DHE fluorescence intensities between AngII-infused and saline-infused p47^phox^ KO hearts. 

### 3.3. AngII-Induced Upregulation of Nox Subunits, PKCα and C-Src Protein Kinases and p47^phox^ Phosphorylation in Murine Hearts

**Figure 3:**
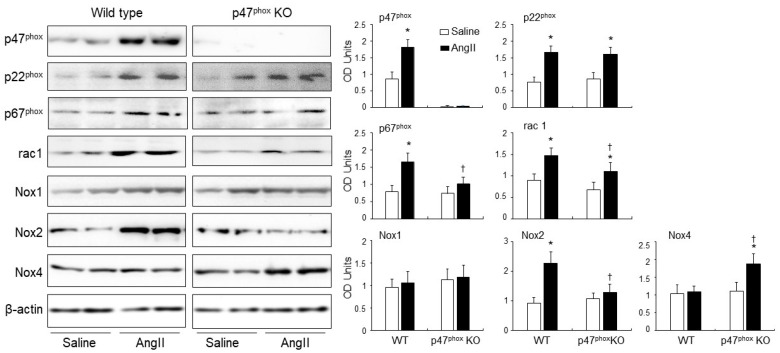
Expressions of isoforms of the catalytic subunit of Nox (i.e., Nox1. Nox2, Nox4) and other subunits of Nox (p47^phox^, p22^phox^, p67^phox^ and rac1) in murine hearts. Left panels: Representative Western blot images. β-actin detected in the same samples were used as loading controls. Right panels: Optical densities (OD) of Western blot bands were quantified and normalised to the levels of β-actin detected in the same samples. *n* = 9 per group. Statistical comparisons were made using two-way ANOVA. * *p* < 0.05 for AngII values versus saline values in the same genetic group; ^†^ *p* < 0.05 for p47^phox^ KO AngII values versus WT AngII values.

The levels of expression of p47^phox^, p22^phox^, p67^phox^, rac1, Nox1, Nox2 and Nox4 in response to AngII infusion were examined in WT and p47^phox^ KO hearts by immunoblotting (Figure 3). The levels of β-actin detected in the same sample were used as loading controls. The p47^phox^ was highly expressed in the WT hearts, but was barely detectable in the p47^phox^ KO hearts. AngII infusion resulted in a great upregulation of the levels of p22^phox^ expression in both WT and p47^phox^ KO hearts without significant difference between the two groups. In comparison to saline infused WT controls, AngII-infusion increased significantly the levels of expression of p47^phox^, p67^phox^, rac1 and Nox2 in the WT hearts. However, in the absence of p47^phox^ KO, AngII infusion had no significant effect on the levels of expression of p67^phox^ and Nox2, but increased significantly the levels of p22^phox^, Nox4 and rac1 expression. Nox1 expression remained the same without significant difference between WT and p47^phox^KO hearts.

**Figure 4:**
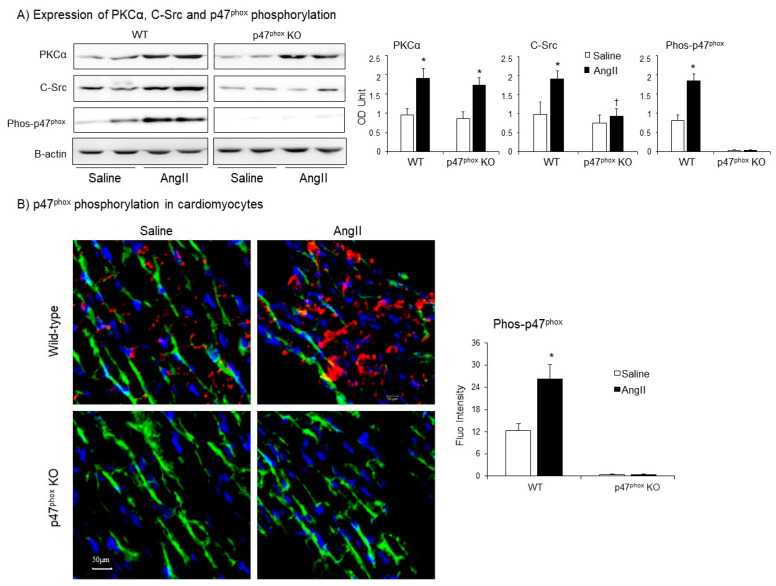
Expressions of protein kinase C alpha (PKCα), Proto-oncogene tyrosine-protein kinase Src (C-Src) and p47^phox^ phosphorylation in AngII-infused murine hearts. (**A**) Western blots. Left panels: Representative images. β-actin detected in the same samples were used as loading controls. Right panels: Optical densities (OD) of Western blot bands were quantified and normalised to the levels of β-actin detected in the same samples. (**B**) Confocal immunofluorescence of cardiac sections. Left panel: Representative immunofluorescence images. Cardiomyocyte cell membrane was labelled by WGA-FITC (green) and p47^phox^ phosphorylation was identified using phos-p47^phox^ specific antibody (Cy3, red). Nuclei were labelled by DAPI (blue). Right panel: Quantification of phos-p47^phox^ fluorescence intensity. *n* = 9 hearts per group. Data were presented as Mean ± SD. Statistical comparisons were made using two-way ANOVA. * *p* < 0.05 for AngII values versus saline values in the same genetic group.

Protein kinase C alpha (PKCα) is highly expressed in the myocardium [22], and phosphorylates p47^phox^ at multiple serine sites in response to AngII stimulation. [23]. C-Src had been proposed to be an upstream tyrosine kinase that phosphorylates p47^phox^ in response to AngII stimulation [24]. Therefore, we examined the levels of expressions of (PKCα) and C-Src together with the levels of p47^phox^ phosphorylation in WT and p47^phox^ KO hearts by immunoblotting (Figure 4A). Compared to saline-infused controls, AngII infusion increased the levels of PKCα expression in both WT and p47^phox^ KO hearts without significant difference between these two groups. However, AngII-induced C-Src expression was only found in WT hearts, but not in p47^phox^ KO hearts suggesting a key role of oxidative stress in cardiac C-Src activation (Figure 4A). Accompanied with increased PKCα expression, there were significant increases in p47^phox^ serine phosphorylation detected using specific antibodies to phos-p47^phox^. 

AngII-induced p47^phox^ phosphorylation in the myocardium was further examined by confocal immunofluorescence (Figure 4B). The sarcolemma membranes of cardiomyocytes were labelled with FITC-WGA (green), and the nuclei were labelled with 4′,6-diamidino-2-phenylindole (DAPI, blue) to visualise cardiomyocytes. The phospho-p47^phox^ was labelled by Cy3 (red), and was only detected in WT hearts. AngII infusion significantly increased the levels of p47^phox^ phosphorylation (red) mainly located at the cardiomyocyte gap junctions or at the cell membranes overlapped with FITC-WGA as indicated by the yellow colour (Figure 4B).

### 3.4. p47^phox^-Dependent Redox-Signalling through MKK3/6, MAPKs and AKT in AngII-Induced and Cardiac Hypertrophy and Apoptosis

**Figure 5:**
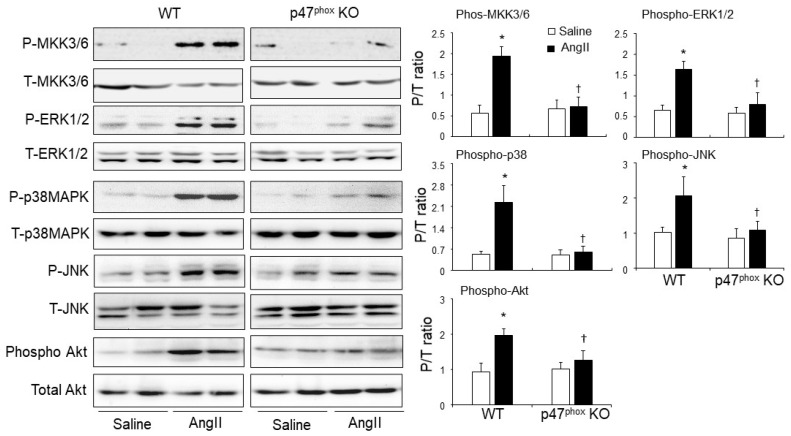
AngII-induced activation of mitogen-activated protein kinase kinase 3/6 (MKK3/6), mitogen-activated protein kinases (i.e., ERK1/2, p38MAPK and JNK) and Akt (also called protein kinase B) in murine hearts. Left panels: Representative immunoblotting images. The total protein bands of each molecule in heart homogenates were pre-tested for equal loading. Right panels: Quantification of the optical densities (OD) of phos-protein bands expressed as phosphorylated/total (P/T) protein ratio. *n* = 9 mice per group. Data were presented as Mean ± SD. Statistical comparisons were made using two-way ANOVA. * *p* < 0.05 for AngII values versus saline values in the same genetic group. ^†^ *p* < 0.05 for p47^phox^ KO AngII values versus WT AngII values.

The role of p47^phox^ in modulating AngII signalling in the hearts was examined for the activations of stress-signalling pathways, i.e., mitogen-activated protein kinase kinase (MKK3/6) and down-stream ERK1/2; p38MAPK, JNK and Akt (Figure 5). The total levels of the same protein in the same samples were pre-tested and used as loading controls. In saline-infused control hearts, there was no significant difference in the levels of phosphorylation of these signalling molecules between WT and p47^phox^KO hearts. Compared to saline-infused controls, AngII-infusion resulted in significant increases in the levels of phosphorylation of MKK3/6, ERK1/2, p38 MAPK, JNK and Akt in WT hearts. However, in the absence of p47^phox^, AngII failed to induce the phosphorylation of these signalling molecules in the hearts (Figure 5).

**Figure 6:**
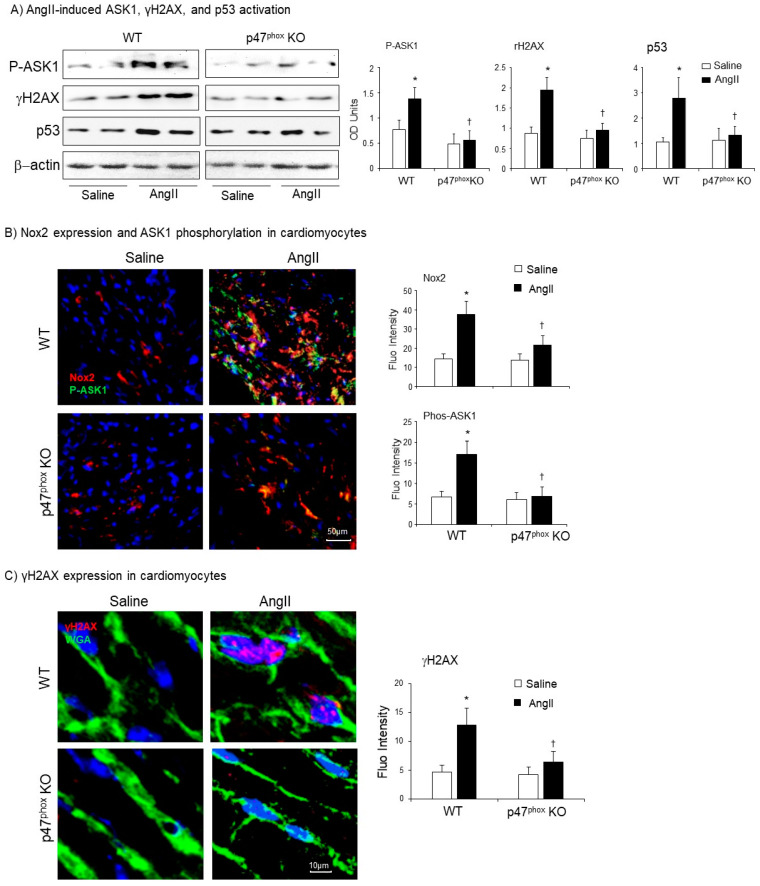
Activation of apoptosis signal-regulating kinase 1 (ASK1), p53 and phosphorylation of H2A histone family member X (γH2AX) in murine hearts. (**A**) Western blots for the expressions of phos-ASK1, p53 and γH2AX. Optical densities of protein bands were quantified and normalised to the levels of β-actin detected in the same samples. (**B**) Confocal immunofluorescence detection of Nox2 (Cy3 labelled, red) and phos-ASK1 expressions (FITC labelled, green) in the cardiac sections. (**C**) Confocal immunofluorescence detection of γH2AX expression (Cy3-labelled, red) in the cardiac sections. The cardiomyocyte membrane was labelled by WGA-FITC (green), and the nuclei were labelled by DAPI (blue). The specific fluorescent densities were quantified. *n* = 9 mice per group. Data were presented as Mean ± SD. Statistical comparisons were made using two-way ANOVA. * *p* < 0.05, for AngII values versus saline values in the same genetic group. ^†^ *p* < 0.05 for p47^phox^ KO AngII values versus WT AngII values.

The effects of genetic knockout of p47^phox^ on AngII-induced oxidative damage of cardiomyocytes and apoptotic death was examine by immunoblotting of apoptosis signal-regulating kinase 1 (ASK1) and biomarkers for DNA double-strand breaks (γH2AX), and apoptosis (p53) (Figure 6A). The levels of β-actin detected in the same sample were used as loading controls. Compared to saline-infused controls, there were remarkable significant increases in the level of expression of phos-ASK1, γH2AX and p53 in AngII-infused WT mice. However, in the absence of p47^phox^, there was no significant increase in the expression of these markers of cell DNA damage and apoptosis in after two weeks of AngII-infusion. 

The crucial role of p47^phox^ in regulating AngII-induced Nox2 activation and ASK1 activation in the cardiomyocytes were examined using immunofluorescence confocal microscopy (Figure 6B). Low levels of Nox2 expression (red) could be detected in the control hearts (infused with saline) without significant difference between WT and p47^phox^KO groups. AngII infusion significantly increased Nox2 expression together with great increases in ASK1 phosphorylation in the WT hearts. AngII-induced Nox2 expression was inhibited in p47^phox^KO hearts and there was no change in the levels of ASK1 phosphorylation in response to AngII infusion in p47^phox^KO hearts. 

The role of p47^phox^ in modulating AngII-induced DNA damage in cardiomyocytes was further demonstrated using immunofluorescence confocal microscopy (Figure 6C). The cardiomyocyte membranes were labelled with FITC-WAG (green), the nuclei were labelled by DAPI. In saline-infused hearts, there was very low level of γH2AX positive staining. However, in AngII-infused WT hearts, there were clear visible γH2AX foci (red) formation detected in the nuclei (blue) of cardiomyocytes as indicated by the pink colour. AngII-induced nuclear expression of γH2A, seen in WT hearts, was significantly inhibited in p47^phox^KO hearts. Putting together, our results indicated clearly a key role of p47^phox^ in mediating AngII-induced oxidative stress, activation of stress signalling pathways and oxidative damage of cardiomyocyte DNAs and cell apoptosis. 

## 4. Discussion

AngII is a potent activator of Nox2 enzyme, which by generating ROS is involved in AngII-induced cardiovascular oxidative stress, hypertension, remodelling and organ damage [2,3]. The p47^phox^ is a primary regulatory subunit of Nox2 enzyme, and the phosphorylation of p47^phox^ at multiple serines in the C-terminus is a key step for Nox2 O_2_^●−^ production [6,17]. However, the signalling pathways of p47^phox^ in the heart remains unclear. The current study by using a disease model of AngII infusion-induced hypertension and cardiac hypertrophy in WT versus p47^phox^KO mice, provided novel insights of p47^phox^-dependent signalling pathways in modulating AngII-induced cardiac hypertrophy and cardiomyocyte apoptosis. We discovered that p47^phox^-dependent regulation of redox-sensitive signalling cascade through ASK1, MKK3/6 and MAPKs is essential in mediating AngII-induced cardiac hypertrophy and DNA damage in cardiomyocytes. Genetic knockout of p47^phox^ inhibited AngII-induced cardiac ROS production, attenuated ASK1, MKK3/6 and MAPK activation and protected cardiomyocyte from AngII-induced hypertrophic growth, DNA damage and apoptosis.

The mice used in this study were 7-month-old, equivalent to humans at the middle-age, and were more susceptible to AngII-induced cardiovascular damages than mice at younger ages. The crucial role of p47^phox^ in mediating AngII-induced cardiac hypertrophy was properly controlled using age-matched littermates of p47^phox^KO mice subjected to the same experimental procedures. Despite a mild increase in BP found in p47^phox^KO mice after two weeks of AngII-infusion, there was no significant cardiac hypertrophy as evaluated using two separate methods, i.e., the changes in HW/BW ratio and cardiomyocyte cross-sectional areas.

NADPH oxidase family contains at least 7 members (Nox1–5 and duox 1–2) [1]. Individual Nox enzyme has distinctive mechanism of activation and functions differently [25,26]. So far, Nox1–2 and Nox4–5 have been found in the hearts [27]. Nox2 relies on p47^phox^ to be active and generates O_2_^●−^ involved in many diseased conditions [6]. Whereas, Nox4 is autoactivated and plays a protective role in cardiovascular function [27]. In the current study, we found that AngII-infusion induced a great increase in cardiac Nox2 expression together with increased level of O_2_^●−^ production in the WT hearts. O_2_^●−^ is short lived and can be quickly converted to H_2_O_2_ by SOD as a cellular self-protective mechanism [28]. This explained the mild elevation of H_2_O_2_ production observed in AngII-infused WT hearts. However, AngII-infusion of p47^phox^KO mice induced a great increase in cardiac Nox4 expression together with a high level of H_2_O_2_ production indicating Nox4 was the enzymatic source of AngII-induced H_2_O_2_ production in p47^phox^KO hearts. 

PKCα has been reported to be highly expressed in the hearts [20] and phosphorylates p47^phox^ at multiple serine sites in response to AngII stimulation [21]. C-Src had also been proposed to be an upstream tyrosine kinase of p47^phox^ phosphorylation in response to AngII stimulation [22]. However, a recent study found that C-Src, rather than an upstream kinase of p47^phox^ phosphorylation, was a downstream molecule of p47^phox^-dependent ROS production in lung inflammation [29]. In the current study, we found that AngII-induced cardiac C-Src activation was oxidant-dependent and was abolished by the knockout of p47^phox^.

MAPKs belong to a highly conserved family of Ser-Thr protein kinases and have diverse regulatory roles in normal heart development as well as in pathological cardiac hypertrophic growth and remodelling [30]. MAPK activation in response to Nox2-derived oxidative stress is a crucial signalling pathway involved in the development of cardiovascular abnormalities. Akt is also a redox-sensitive Ser-Thr kinase involved in cardiomyocyte hypertrophic growth and survival. In the current study, we showed that knockout of p47^phox^ attenuated Nox2-derived ROS production, inhibited MKK3/6, MAPK and Akt activation in response to AngII challenge and protected murine hearts from AngII-induced cardiac hypertrophy. However, p47^phox^ redox-signalling is a complicated mechanism and we do not know if p47^phox^ is physically a component of these signalling pathways, and how it promotes both the pro- and anti-apoptotic signalling pathways in response to AngII infusion. Further detailed investigation is needed.

**Figure 7:**
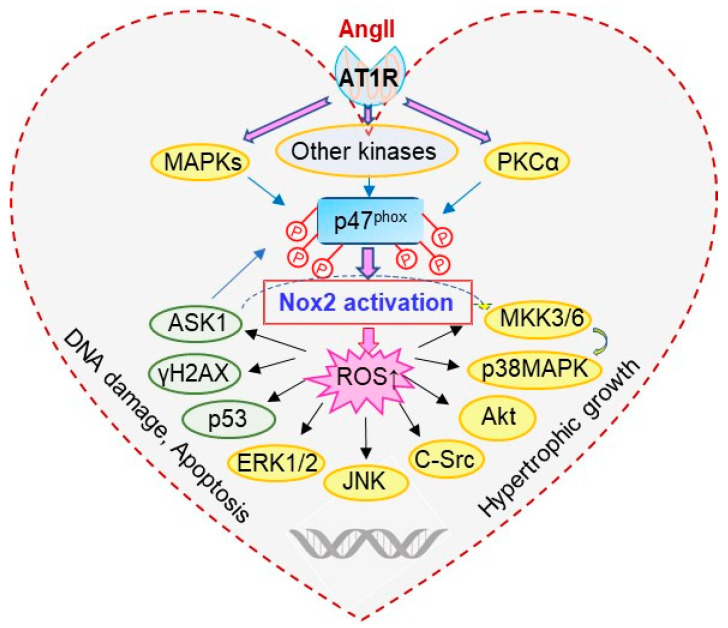
Schematic illustration of p47^phox^ redox-signalling pathways examined.

ASK1 is a member of the mitogen-activated protein kinase kinase kinase (MAPKKK) family that activates downstream MAPKs, JNKs and p38 MAPKs in response to various stresses, such as ROS [13,31]. H2AX is a variant of the H2A protein family and is a component of the histone octamer in nucleosomes [32]. When DNA is damaged and double stranded DNA breaks, H2AX is phosphorylated to form γH2AX. Therefore, γH2AX has been used as a biomarker of DNA damage [32]. The p53 plays an important role in the regulation of cardiomyocyte hypertrophic growth and apoptosis [33]. An important discovery from this study is that ASK1 links p47^phox^ with the activation of MAPKs and the expression of apoptotic markers, i.e., γH2AX and p53, in AngII-induced cardiac hypertrophy and apoptosis. We showed that ASK1 was phosphorylated in response to AngII-induced oxidative stress in the WT hearts. Knockout of p47^phox^, inhibited AngII-induced ASK1 phosphorylation and its down-stream signalling pathways. There was no obvious cardiomyocyte hypertrophic growth and no increase in the expression of apoptosis markers in AngII-infused p47^phox^KO hearts. A schematic illustration of AngII-induced p47^phox^ redox signalling pathways examined in this study is shown in Figure 7. 

## 5. Conclusions

In conclusion, we have reported that p47^phox^ is a key player in mediating AngII-induced oxidative stress signalling cascade from the phosphorylation of ASK1, MKK3/6 and MAPKs to the activation of H2AX and p53 involved in DNA damage and apoptosis of cardiomyocytes. Genetic ablation of p47^phox^ inhibited the cardiac Nox2-derived O_2_^●−^ production, attenuated the activation of ASK1 and MAPK signalling pathways and protected hearts from AngII-induced hypertrophic growth and DNA damage. Targeting p47^phox^ has great therapeutic potential in preventing or treating AngII-induced cardiac dysfunction and damages.

## Data Availability

Research material sources and all data that support the findings of this study are provided within this paper.
